# Genome-Wide Analysis of *NF-Y* Genes in Potato and Functional Identification of *StNF-YC9* in Drought Tolerance

**DOI:** 10.3389/fpls.2021.749688

**Published:** 2021-10-12

**Authors:** Shigui Li, Ning Zhang, Xi Zhu, Rui Ma, Shengyan Liu, Xiao Wang, Jiangwei Yang, Huaijun Si

**Affiliations:** ^1^State Key Laboratory of Aridland Crop Science, Gansu Agricultural University, Lanzhou, China; ^2^College of Agronomy, Gansu Agricultural University, Lanzhou, China; ^3^College of Life Science and Technology, Gansu Agricultural University, Lanzhou, China

**Keywords:** NF-Y transcription factor, potato, bioinformatics, abiotic stresses, root length, stomata closure, drought tolerance

## Abstract

The nuclear factor Y (NF-Y) family is comprised of transcription factors that have been implicated in multiple plant biological processes. However, little is known about this family in potato. In the present study, a total of 41 *StNF-Y* genes were identified in the potato genome. In addition, the phylogenetic, gene structure, motif, and chromosomal location of this family were analyzed. The tissue expression profiles based on RNA-seq data showed that 27 *StNF-Y* genes had tissue-specific expression, while the remaining 14 had low expression in all tissues. Publicly available transcriptomics data from various abiotic stresses revealed several stress-responsive *StNF-Y* genes, which were further verified *via* quantitative real-time polymerase chain reaction experiments. Furthermore, the *StNF-YC9* gene was highly induced by dehydration and drought treatments. StNF-YC9 protein was mainly localized in the nucleus and cytoplasmic membrane. Overexpressing StNF-YC9 potato lines (OxStNF-YC9) had significantly increased in root length and exhibited stronger stomatal closure in potato treated by polyethylene-glycol and abscisic acid. In addition, OxStNF-YC9 lines had higher photosynthetic rates and decreased water loss under short-term drought stress compared to wild-type plants. During long-term drought stress, OxStNF-YC9 lines had higher proline levels, lower malondialdehyde content, and increased activity of several antioxidant enzymes, including superoxide dismutase, catalase, and peroxidase. This study increased our understanding of the *StNF-Y* gene and suggested that *StNF-YC9* played an important role in drought tolerance by increased the photosynthesis rate, antioxidant enzyme activity, and proline accumulation coupled to lowered malondialdehyde accumulation in potato.

## Introduction

Nuclear factor Y (NF-Y) is a ubiquitous transcription factor family that is found in almost all eukaryotes (Myers and Holt, 2018). NF-Y transcription factors are also known as CCAAT-binding factors (CBFs) and heme-associated proteins (HAPs). They consist of three distinct families, including NF-YA (CBF-B/HAP2), NF-YB (CBF-A/HAP3), and NF-YC (CBF-C/HAP5; Nardini et al., 2013). In mammals and yeasts, each NF-Y subunit is encoded by a single gene (Li et al., 1992), while plant genes encoding these subunits have expanded (Petroni et al., 2012), enabling the acquisition of new molecular functions. Thus far, 36 NF-Y genes have been identified in Arabidopsis (*Arabidopsis thaliana*; Siefers et al., 2009), 34 in rice (*Oryza sativa*; Yang et al., 2017), 40 in chickpea (*Cicer arietinum*; Chu et al., 2018), and 42 in poplar (*Populus trichocarpa*; Liu et al., 2020).

NF-Y subunits have been shown to be comprised of two conserved *α*-helices, one of which is located at the N-terminus (A1), while the other is located at the C-terminus (A2). The A1 helix plays a role in protein interaction, while the A2 helix provides sequence specificity for binding to CCAAT sites (Frontini et al., 2004; Laloum et al., 2013). NF-YB/C subunits contain a highly conserved histone fold domain (HFD) that is typically comprised of three *α*-helices (a1, a2, and a3) and another *α*-helix domain at the C-terminal region (Petroni et al., 2012). These structures determine the NF-YB and NF-YC subunit specificity in protein-DNA and protein-protein interaction (Kahle et al., 2005). The NF-B6/NF-YC3 crystal structure in Arabidopsis has been resolved, which revealed that AtNF-YB6 and AtNF-YC3 are arranged in a head-to-tail orientation (Gnesutta et al., 2017; Nardone et al., 2017). Since the CCAAT box has been reported to be present in 25–30% of all eukaryotic gene promoters, the NF-Y family likely plays multiple roles, including growth and development, flowering time, photosynthesis, stress response, signal transduction, and nodulation (Mantovani, 1998; Zhao et al., 2016; Myers and Holt, 2018). In addition, NF-Y members have been reported to participate in fruit ripening (Yan et al., 2019), adaptive responses to nutrient deprivation (Zhao et al., 2011), primary metabolism (Li et al., 2015), and disease resistance (He et al., 2020). In Arabidopsis, *AtNF-YA5* has been shown to be highly expressed under both drought and abscisic acid (ABA) treatment, and overexpression of this gene results in reduced leaf water loss and better drought tolerance (Li et al., 2008). In addition, *AtNF-YA2/3/7/10* have been shown to be induced by drought stress (Leyva-González et al., 2012), while overexpression of *OsNF-YA7* in transgenic rice plants increased drought tolerance by modulating gene regulation in an ABA-independent manner (Dong-Keun et al., 2015). Numerous studies have demonstrated the role of NF-YB subunits in drought stress tolerance (Nelson et al., 2007; Han et al., 2013; Wang et al., 2018). The *CdtNF-YC1* gene was isolated from Bermuda grass, and its overexpression in rice and seashore paspalum was found to result in enhanced drought and salinity tolerance (Chen et al., 2015; Wu et al., 2018). In addition, four *miR169* isoforms (d, e, f, and g) have been shown to work in concert with *NF-YA2* to control root growth and development in Arabidopsis (Sorin et al., 2014). Recently, a *PdNF-YB21* was isolated from poplar that shown to have root-specific expression and positively regulate root growth, resulting in increased drought tolerance (Zhou et al., 2020).

Potato is the fourth largest food crop, and its production is constantly affected by abiotic stresses that are worsening with climate change. Traditional potato breeding is difficult, due to the complex polysomic genetics and heterozygosity of this species (Slater et al., 2014). Therefore, molecular approaches have typically been employed to identify genetic engineering targets to increase potato environmental stress tolerance. A previous study reported that overexpression of *StNF-YA* enhanced drought tolerance in potato (Na et al., 2017). In addition, overexpression of *StNF-YB3.1* was shown to promote ABA-mediated stomatal closure, which resulted in lower tuber production (Xuanyuan et al., 2017). Van Muijen et al. (2016) utilized gene co-expression analysis and network topology to identify a *StNF-YC* gene that is a key genetic determinant of drought-induced gene regulation in diploid potato. Indeed, the NF-Y transcriptions require the formation of heterotrimers conformed of NF-A, NF-YB, and NF-YC subunits or other proteins to exert the function (Petroni et al., 2012). To date, no comprehensive study of the *NF-Y* gene family has been conducted in potato, and the underlying mechanisms of *StNF-YC* genes remain largely elusive.

In the present study, 41 putative *StNF-Y* genes were identified in potato and subjected to phylogenetic, gene structure, motif, and chromosomal location analyses. In addition, the tissue-specific expression profiles, as well as differential expression profiles of potato *StNF-Y* genes under different abiotic stresses, were analyzed. Moreover, OxStNF-YC9 lines under the control of the cauliflower mosaic virus 35S promoter (CaMV 35S) were generated in order to identify the function of StNF-YC9 in the root length and drought tolerance. Our results provide a platform for the further investigation of the functions of *StNF-Y* genes in potato drought stress responses.

## Materials and Methods

### Plant Growth Conditions and Treatments

Potato (*Solanum tuberosum* L. cv. “Desiree”) seedlings were propagated *in vitro* on Murashige-Skoog (MS) medium with 2% sucrose and 0.6% agar in an illuminated incubator maintained 22°C, under a 16-h light / 8-h dark cycle. After 20days of growth *in vitro*, seedlings were transplanted to plastic pots (10cm×10cm) that contained in a 3:1 (v/v) mixture of peat moss and nutrient soil (soil/vermiculite/perlite as the volume proportion of 3.5:1:1) in a growth chamber at 24°C under light of 160μmolm^−2^ s^−1^, with a 16-h light/8-h dark photoperiod and 60% relative humidity. Plants were grown for 28days, followed by the selection of plants with similar height and health for abiotic stress treatments. The leaves were detached and used for gene expression analysis. For osmotic treatment, the plants were irrigated with a solution 300ml of 20% polyethylene-glycol-6000 (PEG-6000). For salt stress, the plants were irrigated with 200mM NaCl (300ml per pot) that the average soil conductivity was 6.37 mS/cm. For dehydration treatment, the detached leaves were placed on a plate in a growth chamber with temperature of 24°C under light of 160μmolm^−2^ s^−1^ and 60% relative humidity. Take the above different treatments for 0, 0.5, 1, 3, 5, 6, and 12h. Water was withheld from the plants for 0, 1, 3, 5, 7, and 9days to simulate drought stress. The samples were frozen in liquid nitrogen immediately and then preserved at −80°C for further use.

### Identification, Phylogenetic Tree Construction, Gene Structure, Motif Analysis, and Multiple Alignment of NF-Y Protein

Arabidopsis AtNF-Y family members were obtained using data from a previous study (Siefers et al., 2009). StNF-Y family members were queried by BLAST search and HMMER (version 3.0) software. AtNF-Y family members were used for BLASTP searches in the Phytozome database.^1^ The potato proteins from each BLAST search were identified and redundant sequences were removed. All the sequences were inspected using the InterPro^2^ and Pfam^3^ databases to confirm the presence of the conserved NF-Y domains. The molecular weights (Mw), isoelectric points (pI), and grand average of hydropathy (GRAVY) were predicted by ExPASy.^4^ Alignments of amino acid sequences of full-length NF-Y proteins were performed by Clustal X1.8. The phylogenetic tree was generated by MEGA 7.0 using the neighbor-joining method with 1,000 bootstrap replicates. The exon and intron distributions of potato StNF-Ys were obtained by GSDS online software.^5^ The conserved motifs of the predicted StNF-Y proteins were analyzed by the program Multiple Em for Motif Elicitation (MEME).^6^

### *StNF-Y* Gene Expression Analysis by Quantitative Real-Time Polymerase Chain Reaction

First-strand cDNA synthesis was performed using the FastKing RT Kit with gDNase (Tiangen Biotech, Beijing). Super Real PreMix Plus (SYBR Green; Tiangen Biotech, Beijing) was used to analyze the expression levels of *StNF-Ys* with gene-specific primers (Supplementary Table S1). The PCR solution (20μl) contained 10μl of 2×Super Real PreMix Plus, 0.6μl of forward and reverse primers, 1μl of cDNA (100ng) template, and 7.4μl of nuclease-free water. qRT-PCR was conducted in a Light Cycler 96 system (Roche, Diagnostics GmbH), with the following parameters: 95°C for 15min, followed by 40cycles of 95°C for 10s and 60°C for 20s. The *Stef1ɑ* gene was used as a reference gene for normalization. The primer sequences are shown in Supplementary Table S1. All experiments were performed with three biological replicates and three technical replicates. The relative expression levels of genes were calculated by the 2^−ΔΔCt^ method (Livak and Schmittgen, 2001).

### Cloning and Subcellular Localization of *StNF-YC9*

The coding sequence of *StNF-YC9* was cloned from the cDNA of potato cv. “Desiree.” The gene-specific primers *StNF-YC9-F1* and *StNF-YC9-R1* (Supplementary Table S1) were designed using DNAMAN 8.0. The PCR fragments were then inserted into the pMD18-T vector (Takara Bio, Beijing) for further sequencing. The coding sequence of *StNF-YC9* without the stop codon was amplified by PCR, using the gene-specific primers *StNF-YC9-F2* and *StNF-YC9-R2* (Supplementary Table S1). The validated PCR product was linked into the vector pCAMBIA1300-35S-EGFP at the site of *Bam*H I and *Sal* I. The plasmid pCAMBIA1300-35S-StNF-YC9-EGFP was transformed into the *Agrobacterium tumefaciens* LBA4404 strain. The pCAMBIA1300-35S-StNF-YC9-EGFP was injected into tobacco leaves (*Nicotiana benthamiana* L.) that were 5–6weeks old. EGFP fluorescence was observed using a laser scanning confocal microscope (CARI ZEISS, LSCM 800, Germany).

### Vector Construction and Generation of Transgenic Potato Plants

The coding sequence of *StNF-YC9* was inserted into the expression vector pCAMBIA1300-35S between *Bam*H I and *Sal* I restriction sites, which was transformed into microtubers *via* the method described by Si et al. (2003). The microtuber was cut by a sterile blade into pieces that were 0.4–0.6cm thick. The microtuber pieces were then placed into a flask filled with 50ml *Agrobacterium* liquid that contained the recombinant plasmid pCAMBIA1300-35S-StNF-YC9 for 7–10min. The microtuber pieces infected with *Agrobacterium* then were transferred to sterile filter paper to absorb the *Agrobacterium* liquid on the surface of the microtuber pieces. Next, the microtuber pieces were placed onto solid MS medium and co-cultivated in the dark for 2days at 28°C. After 2days, the microtuber pieces were transferred onto MS medium for further culturing. Potato plants were screened using selection medium containing 5μgml^−1^ Hygromycin B (Hyg), followed by genomic DNA extraction *via* the CTAB method. Gene-specific primers were used to amplify the 809-bp hygromycin B phosphotransferase (HPT) gene fragment. In addition, qRT-PCR was used to determine the expression level of *StNF-YC9* in the transgenic potato plants.

### Morphological Characterization of the Transgenic Potato Plants

The wide-type (WT) and OxStNF-YC9 potato seedlings were planted *in vitro* on MS agar medium at pH5.8 in an illuminated incubator maintained at 16-h white fluorescent light and 8-h darkness at a temperature of 22°C. Two-week-old potato seedlings were used for morphological characterization. Plant height (the distance from root neck to the top of the plant) and root length were measured by a ruler. The fresh weight and the weight of underground plant portion were measured by an electronic balance.

### Stomatal Aperture Analysis

Leaves were detached from 28-day-old WT and OxStNF-YC9 plants, perforated at the same position on both sides of the main vein with a perforator. The detached leaves were immersed in stomata opening solution that contained 15mM KCl, 10mM CaCl_2_, and 10mM MES-KOH for 3-h in light conditions and then exposed to 10μM ABA for 1h and 2h, and 10% PEG for 24h. The abaxial epidermal layers of the leaves were sampled for microscopy. The stomata images were captured by a Olympus DIC microscope (Olympus, BX61, Japan).

### Drought Stress Treatment

Potato seedlings were propagated *in vitro* on MS agar medium, followed by transplantation to plastic pots (10cm×10cm). After 4weeks of growth under well-watered conditions in an illuminated incubator (light cycle: 16.0-h light, 8.0-h dark; temperature: 22°C), healthy plants were subjected to water deficit stress, by keeping the soil RWC at 45% (mild drought stress) for 7 and 14days, respectively. We weighed the containers daily and supplemented lost water. Three containers without plants were used to estimate the evaporation from soil based on the soil relative water content (RWC). The containers were weighed after being watered to saturation (initial weight). The soil RWC was calculated as (fresh weight−dry weight)/(initial weight−dry weight)×100.

### Measurement of Photosynthesis, Transpiration, Stomatal Conductance, Relative Water Content, and Leaf Water Loss

An open infrared gas analysis system (Li-COR6400, Lincoln, NE, USA) was used to measure the net photosynthesis rate, stomatal conductance, and transpiration rate in OxStNF-YC9 and WT potato plants. Photosynthetic measurements were performed under a photon flux density of 1,200μmolm^−2^ s^−1^, at 25°C and 380μmolmol^−1^ CO_2_. The RWC of leaves was measured by the method of You et al. (2019). The third to fifth fully developed leaves were used for RWC measurements. Leaf water loss was determined by air dyeing. The excised leaves were placed at 22°C and 60% humidity, and water loss was calculated as the percentage of initial fresh weight.

### Measurement of Superoxide Dismutase, Peroxidase, Catalase, Proline, and Malondialdehyde

SOD enzyme activity was determined according to Owens (1985). Briefly, 20μl of enzyme extract was added to a 3-ml reaction mixture of 50mM sodium phosphate (pH7.8), 130mM methionine, 750μM nitro-blue tetrazolium, 100μM EDTA-Na_2_, 20μM riboflavin and distilled water in a ratio of 15:3:3:3:3:2.5, and illuminated under a light intensity of 4,000lx for 30min. Absorbance at 560nm was then measured. POD and CAT activity were analyzed according to the method described by Maehly and Chance (1954). MDA content was determined as described by Heath and Packer (1968), and proline content was measured according to the method described by Bates et al. (1973). Three biological repeats were set up for each experiment.

### Experimental Replication and Statistical Analysis

For treatments on potato seedlings that used for qRT-PCR, different conditions were prepared with three biological replications. A total of 40 (10 per line) potato seedlings were used for the analysis of morphological characterization of OxStNF-YC9 and WT plants. For stomatal aperture analysis, a total of 16 leaves from four plants of WT and transgenic lines were collected, and 80 stomata from each line were randomly selected and measured to calculate stomatal aperture (the ratio of width to length) for statistical analysis. A total of 24 plants (six per line) were measured for net photosynthesis rate, stomatal conductance, and transpiration rate. For the leaf RWC, 10 leaves of each line were determined. In the dehydration on potato, five independent plants were used to measure the fresh weight. The entire experiment was repeated three times.

Data were analyzed by SPSS 22.0 (IBM, Chicago, IL, USA) for statistical testing. All data, with the exception of plant morphological measurements, were statistically analyzed by Duncan’s multiple comparisons test, with all results subjected to Student’s *t* tests.

## Results

### Identification of NF-Y Subunits in Potato

*StNF-Ys* could be divided into three subfamilies, including 10 *StNF-YAs*, 22 *StNF-YBs,* and 9 *StNF*-YCs (Supplementary Table S2). The proteins encoded by the *StNF-YA* genes ranged from 196 (StNF-YA8) to 311 (StNF-YA1) amino acids (aa) in length, while their molecular weights ranged from 22.06 (StNF-Y A8) to 33.97 (StNF-YA1) KDa and their isoelectric points (pIs) ranged from 6.5 (StNF-YA2) to 9.65 (StNF-YA6; Supplementary Table S2). StNF-YB proteins ranged from 135 (StNF-YB6) to 224 aa (StNF-YB9), with 13.82 (StNF-YB18) to 25.03 KDa (StNF-YB9) molecular weights and 4.64 (StNF-YB22) to 9.32 (StNF-YB15) pIs. In the StNF-YC subfamily, the protein lengths, molecular weights (KDa), and pIs ranged from 141 (StNF-YC7) to 293 (StNF-YC8), 15.67 (StNF-YC7) to 32.28 KDa (StNF-YC8), and 4.89 (StNF-YC3) to 9.33 (StNF-YC8), respectively.

### NF-Y Protein Multiple Sequence Alignments, Phylogenetic Analyses, Gene Structures, Motifs, and Chromosomal Location Analyses

**Figure 1:**
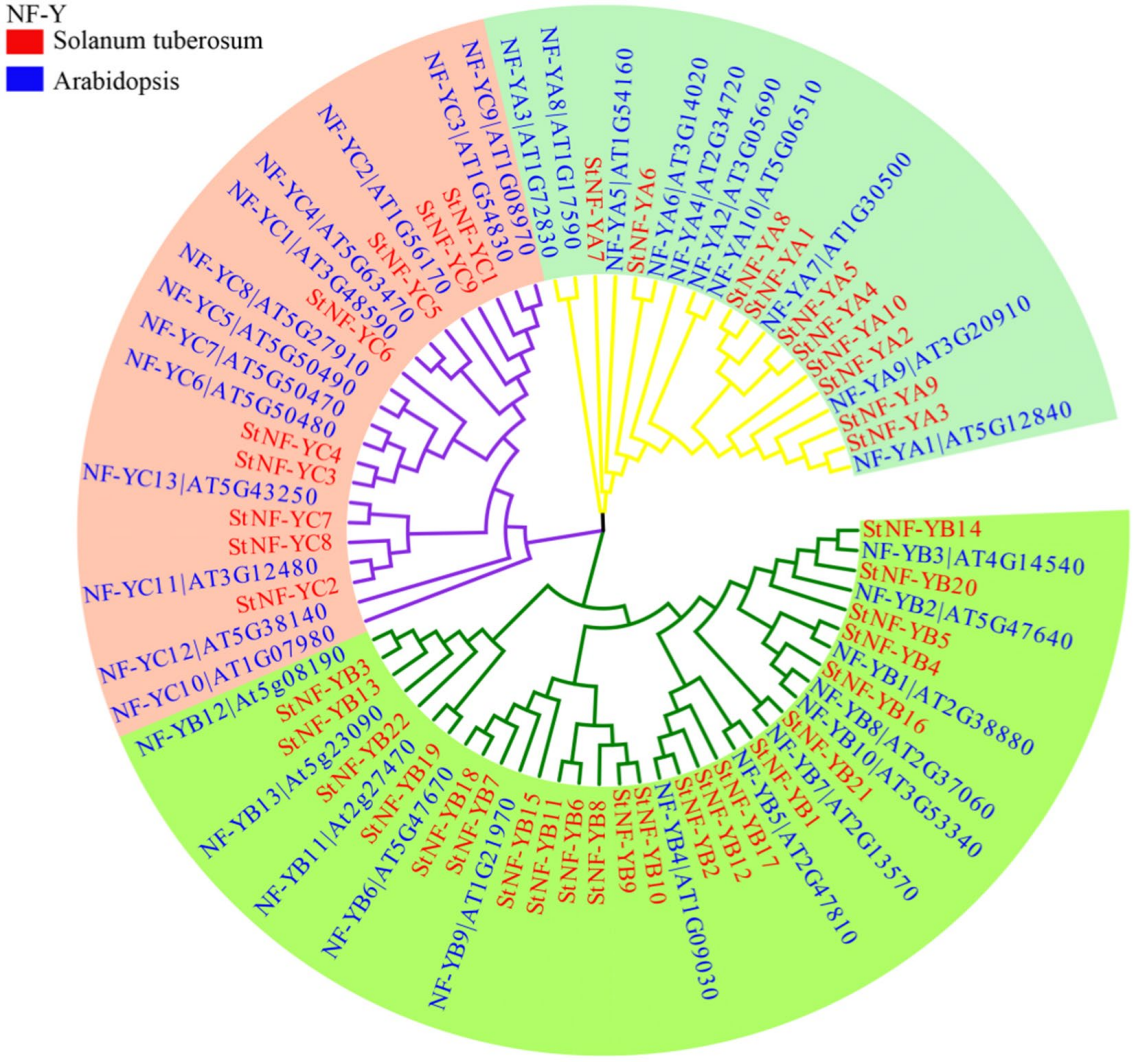
The neighbor-joining phylogenetic tree of NF-Y proteins in potato and Arabidopsis. The phylogenetic tree was constructed by Clustal X 1.8 and MEGA 7.0 software using the neighbor-joining option with 1,000 bootstrap replicates. The red rectangle and blue rectangle represent the StNF-Y and AtNF-Y protein, respectively. Branch lines in different colors represented different subgroups.

**Figure 2:**
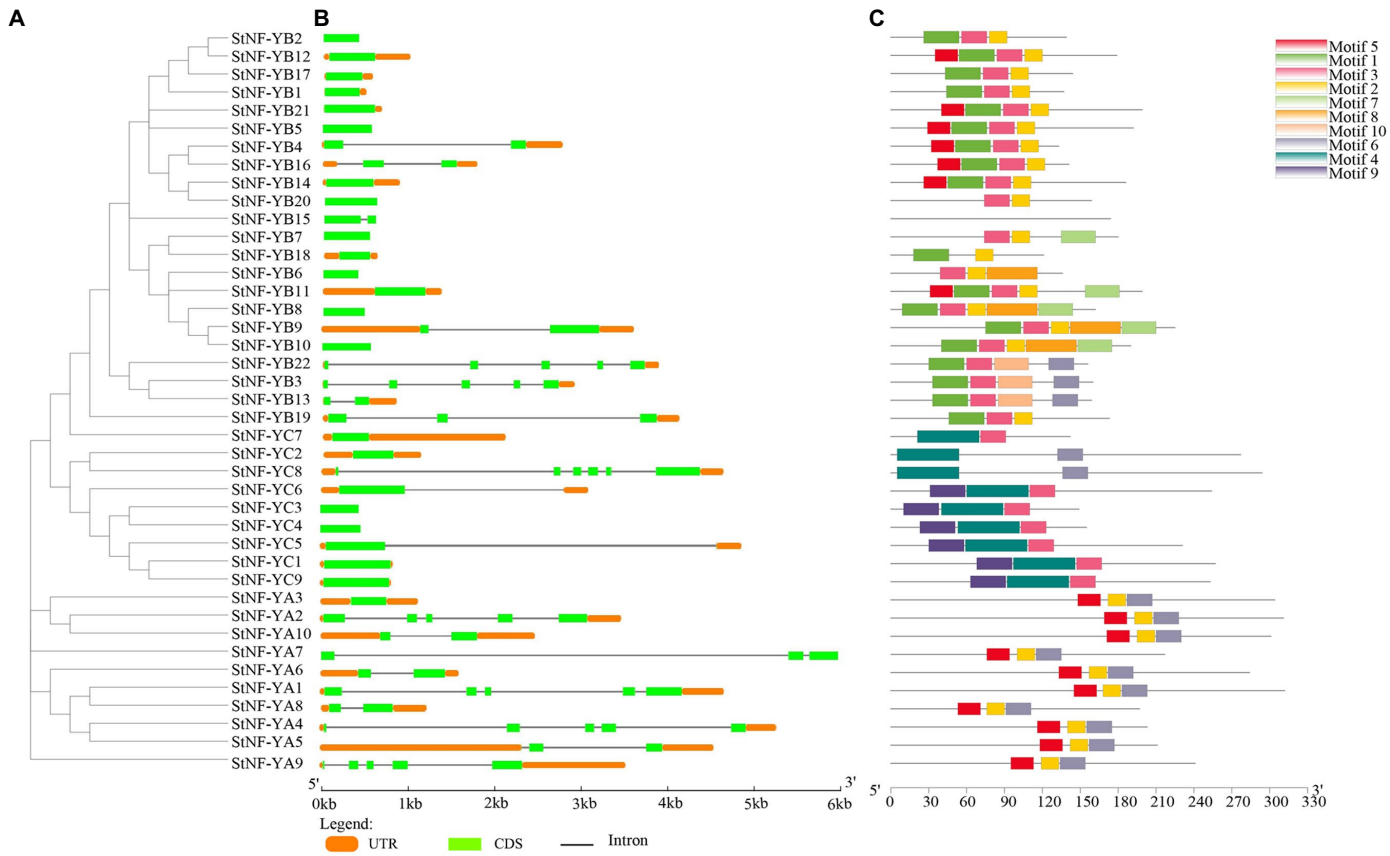
Phylogeny, gene structure, and distribution of conserved motifs in StNF-Y proteins. **(A)** The phylogenetic tree was built on the basis of the full amino acids of 41 StNF-Y proteins by the neighbor-joining method implemented by MEGA software. **(B)** Gene structures of the 41 potato *NF-Y* genes. The exons and introns were indicated by light green rectangles and thin lines, respectively. The untranslated regions (UTRs) were indicated by orange rectangles. **(C)** Motifs of the StNF-Y proteins were identified using the online MEME program. Different colors boxes represented different motifs, for motif details were listed in Supplementary Table S3.

The results of the multiple sequence alignments of the potato and *A. thaliana* NF-Y subunits indicated that each subfamily was found to have one or more central core regions with extensive homologous motifs (Supplementary Figure S1). To understand the evolutionary relationship between StNF-Y family and AtNF-Y family, an unrooted tree was constructed based on an alignment of the 41 identified StNF-Y and 36 AtNF-Y with conserved domain sequences by MEGA7.0 software using the NJ method. All 41 StNF-Y proteins were distinctly classified into three major groups (NF-A, NF-YB, and NF-YC; Figure 1). The evolutionary relationship suggested that the NF-Y protein family in *A. thaliana* has similar structure and function to that in potato. To better understand the *StNF-Y* genes, their gene structures were analyzed (Figure 2). In the *StNF-YA* subfamily, four members (*StNF-YA1/2/4/9*) were found to have five exons, while four members (*StNF-YA5/6/8/10*) had two exons. Fourteen *StNF-YB* genes had no intron, while *StNF-YB3/22* had four introns. In addition, *StNF-YB2/5/6/7/8/10/15/20* lacked untranslated regions (UTRs). All *StNF-YC* genes contained one exon, except for *StNF-YC8*, which had six exons. Within the different subfamilies, genes tended to have similar gene structures, including exon length, exon number, and total gene length. A total of 10 different conserved motifs were found in the StNF-Y proteins (Figure 2; Supplementary Table S3). Motifs 2, 5, and 6 were present in the StNF-YA subfamily, and motif 2 was found to be similar to a DNA binding domain. Motifs 1, 2, 3, and 5 were found to be similar to histone-like transcription factor domains (CBF/NF-Y) and were exclusively found in the StNF-YB subfamily. Motif 4 was unique to the StNF-YC subfamily. Moreover, StNF-Y members within the same subgroups were universally found to share common motifs. *StNF-Y* genes were found on all 12 chromosomes, with chromosomes one and five possessing the highest number (seven; Supplementary Figure S2).

### Expression Patterns of *StNF-Y* Genes in Different Tissues and in Response to Abiotic Stresses

**Figure 3:**
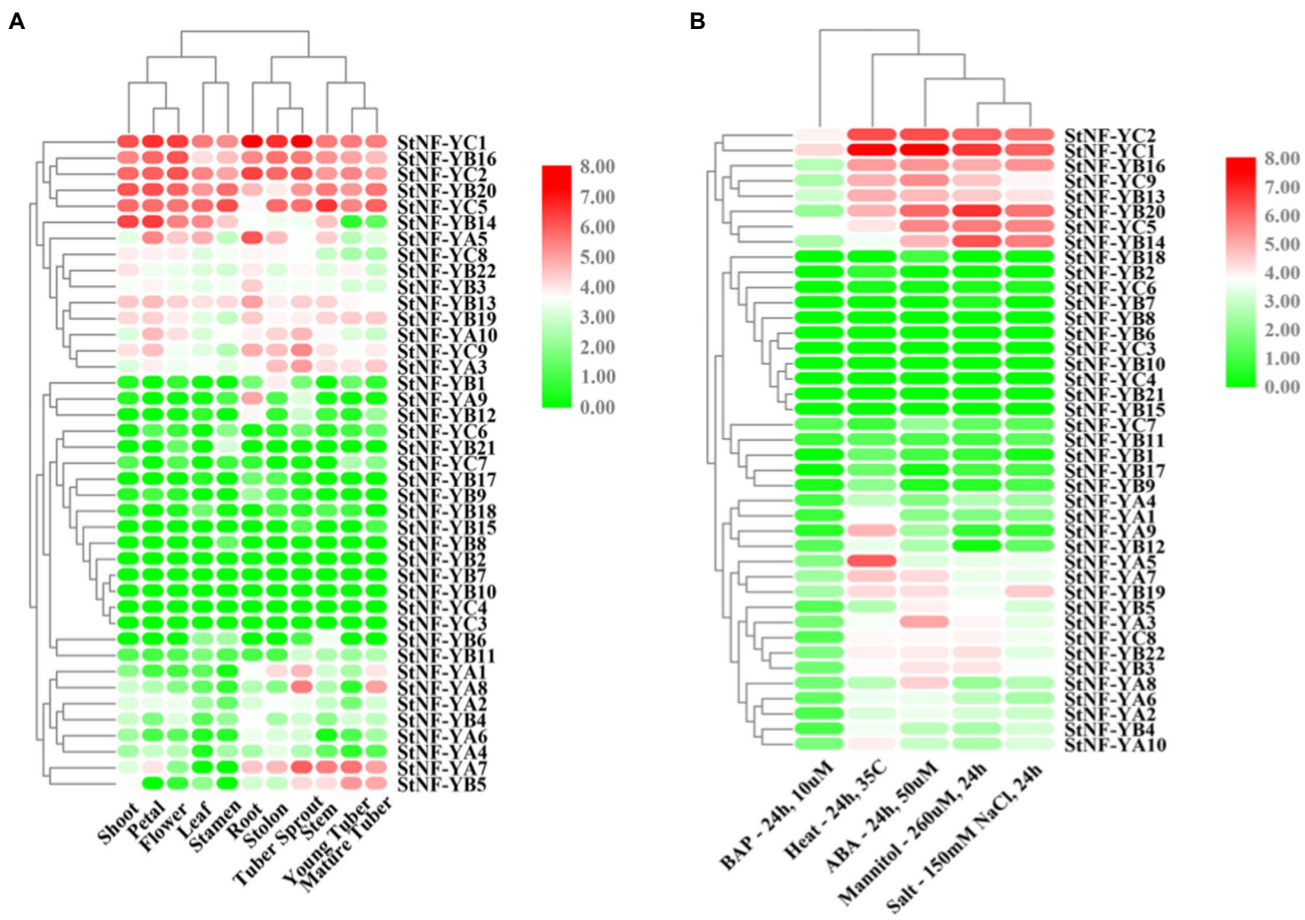
Expression patterns of 41 *StNF-Y* genes in various potato organs and in different stress conditions. **(A)** The expression profiles of *StNF-Y* genes in 11 different potato organs and tissues. **(B)** The expression profiles of *StNF-Y* genes under five different stress treatments. RNA-seq expression data corresponding to 41 potato *NF-Y* genes were retrieved from the international potato genome sequencing consortium (PGSC) data sets for further analysis. The heat map was generated based on the RPKM (Reads Per Kilobase of exon model per Million mapped reads) values that were transformed to log2 (value +1). Red and green color gradients indicate an increase or decrease, respectively.

**Figure 4:**
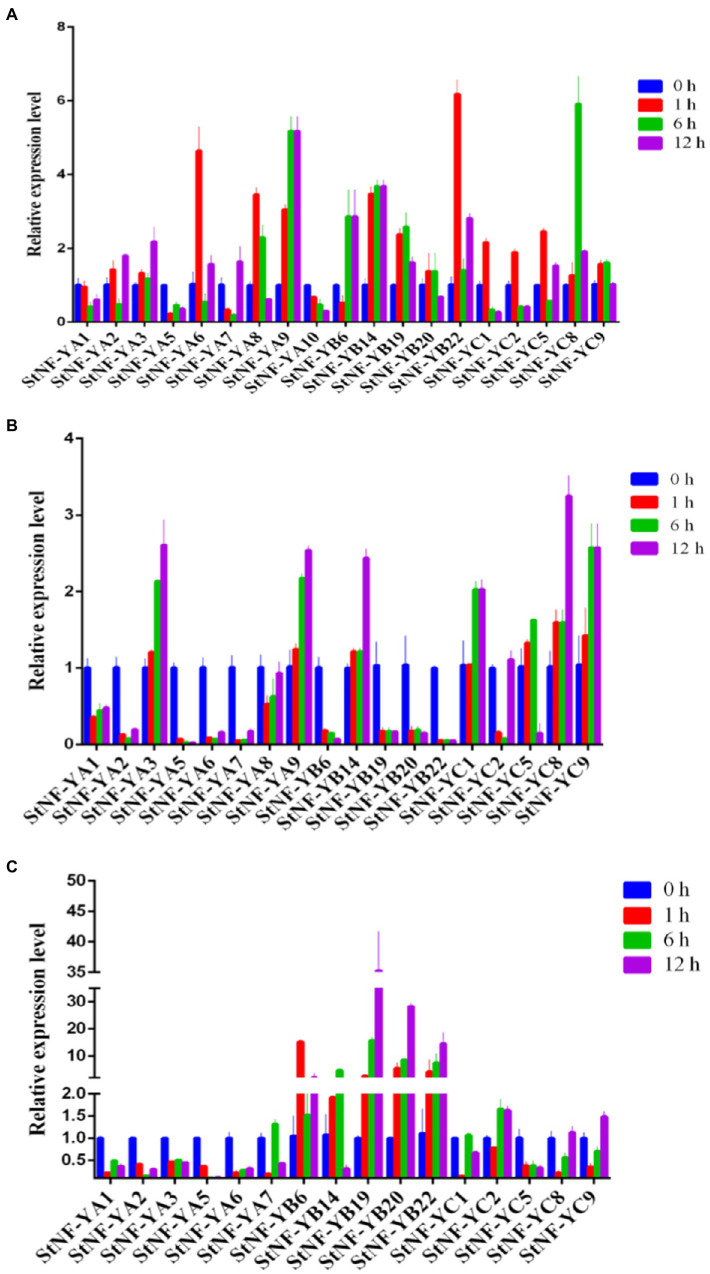
Expression profiles of *StNF-Y* genes following treatment of 28-day-old seedlings with salt **(A)**, drought **(B)**, and dehydration **(C)**. The transcript levels of each *StNF-Y* in the stress-treated plants (1, 6, and 12 h treatment) were plotted as the relative expression of the untreated control plants. Mean values and standard deviations (SDs) were obtained from three biological and three technical replicates.

To explore the possible functions of *StNF-Y* genes in potato development, the expression patterns of 41 *StNF-Y* genes in different tissues, including shoots, petals, flowers, leaves, stamens, roots, stolons, tuber sprouts, stems, young tubers, and mature tubers, as well as during different stresses, were assessed *via* RNA-seq data. Five *StNF-Y* genes (*StNF-YC1*/*2*/*5* and *StNF-YB16*/*20*) were highly expressed in all organs surveyed, while 13 *StNF-Y* genes (*StNF-YB2/6/7/8/9/10/11/15/18* and *StNF-YC3/4/6/7*) were present at a low level of expression in all tissues. *StNF-YA8* was found to be primarily expressed in mature tubers and tuber sprouts, while *StNF-YA9* was specifically expressed in roots (Figure 3A). The variable expression patterns of *StNF-Ys* suggested a divergence in biological functions during potato growth and development. Additionally, a total of eight *StNF-Y* genes (*StNF-YC1/2/5/9* and *StNF-YB13/14/16/20*) were highly expressed under the heat, mannitol, and ABA treatment conditions, while 15 genes (*StNF-YB1/2/6/7/8/9/10/11/15/17/18* and *StNF-YC3/4/6/7*) were expressed at a low level under all stress treatments (Figure 3B). To verify the stress-specific expression of *StNF-Y* genes from the RNA-seq data, 29 *StNF-Y* genes were subjected to qRT-PCR confirmation in potato leaves treated with salt, drought, dehydration, or ABA stresses. During salt stress, the expression of *StNF-YA9/B14/22/C8* genes was upregulated (Figure 4A), with *StNF-YB14* closely recapitulating the RNA-seq results (Figure 3B). During 20% PEG-6000 stress, two genes (*StNF-YA3/9/B14/C1/8/9*) were upregulated, while the other selected candidate genes (*StNF-YA1/2/5/6/7/8/B6/19/20/22*) were downregulated (Figure 4B). Dehydration resulted in increased expression for *StNF-YB19/20/22*, whereas *StNF-YA1*/*2/3/5/6* genes were downregulated (Figure 4C).

### Tissue Expression Patterns and Subcellular Localization of StNF-YC9

**Figure 5:**
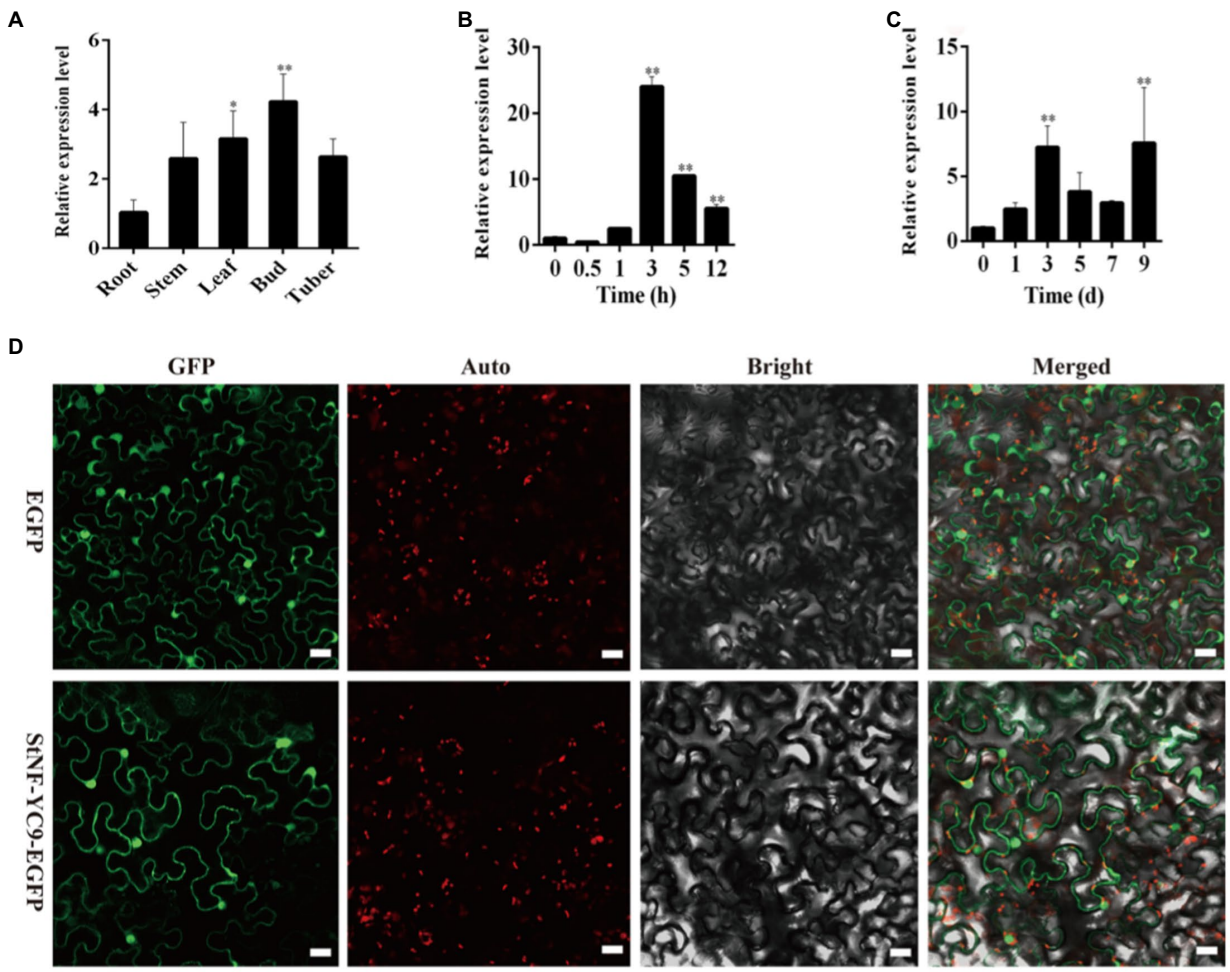
Analysis of potato StNF-YC9. **(A)** The expression level of *StNF-YC9* in various organs of potato. **(B)** The expression level of *StNF-YC9* in potato leaves under dehydration stress. **(C)** The expression level of *StNF-YC9* in potato leaves under drought stress. **(D)** Subcellular localization of StNF-YC9 protein in tobacco leaf cells. The EGFP and StNF-YC9-EGFP fusion protein transiently expressed in tobacco. The scale bale represents 20μm. The images contain green fluorescent protein field, chlorophyll auto-fluorescent signal, bright field, and merged microscope images. The asterisks represent significant differences compared to the control (^*^p<0.05 and ^**^p<0.01).

To understand the tissue-specific expression patterns of *StNF-YC9* in potato, root, stem, leaf, bud, and tuber tissues were used to quantify its expression under control conditions. *StNF-YC9* was found to be expressed more highly in buds, leaves, and tubers than in stems and roots (Figure 5A). Additionally, *StNF-YC9* expression was found to be induced by dehydration, peaking at 3h after the initiation of stress conditions (Figure 5B). When subjected to a 9-day drought treatment, the expression of the *StNF-YC9* gene fluctuated, reaching a peak of 7.53-fold higher than under control conditions (Figure 5C). Transient expression assays in epidermal cells of tobacco showed that StNF-YC9-EGFP fusion protein was mainly expressed in the nucleus and cytoplasmic membranes and might express in the cell wall (Figure 5D), and there is no specific compartmentalization.

### Morphological Characterization of OxStNF-YC9 Plants

Based on the qRT-PCR results (Supplementary Figure S3), the three OxStNF-YC9 lines that showed the highest expression level were selected for further study. The OxStNF-YC9 and WT plants were grown for 14days on MS medium and used for morphological characterization. The roots of the three OxStNF-YC9 lines were 1.43-fold to 1.52-fold longer than that of the WT plants (Supplementary Figure S4; Supplementary Table S4). However, there were no significant differences between the OxStNF-YC9 plants and WT for plant height, fresh weight, root fresh weight, and root to shoot ratio (Supplementary Table S4).

### StNF-YC9 Promotes PEG- and ABA-Induced Stomatal Closure

**Figure 6:**
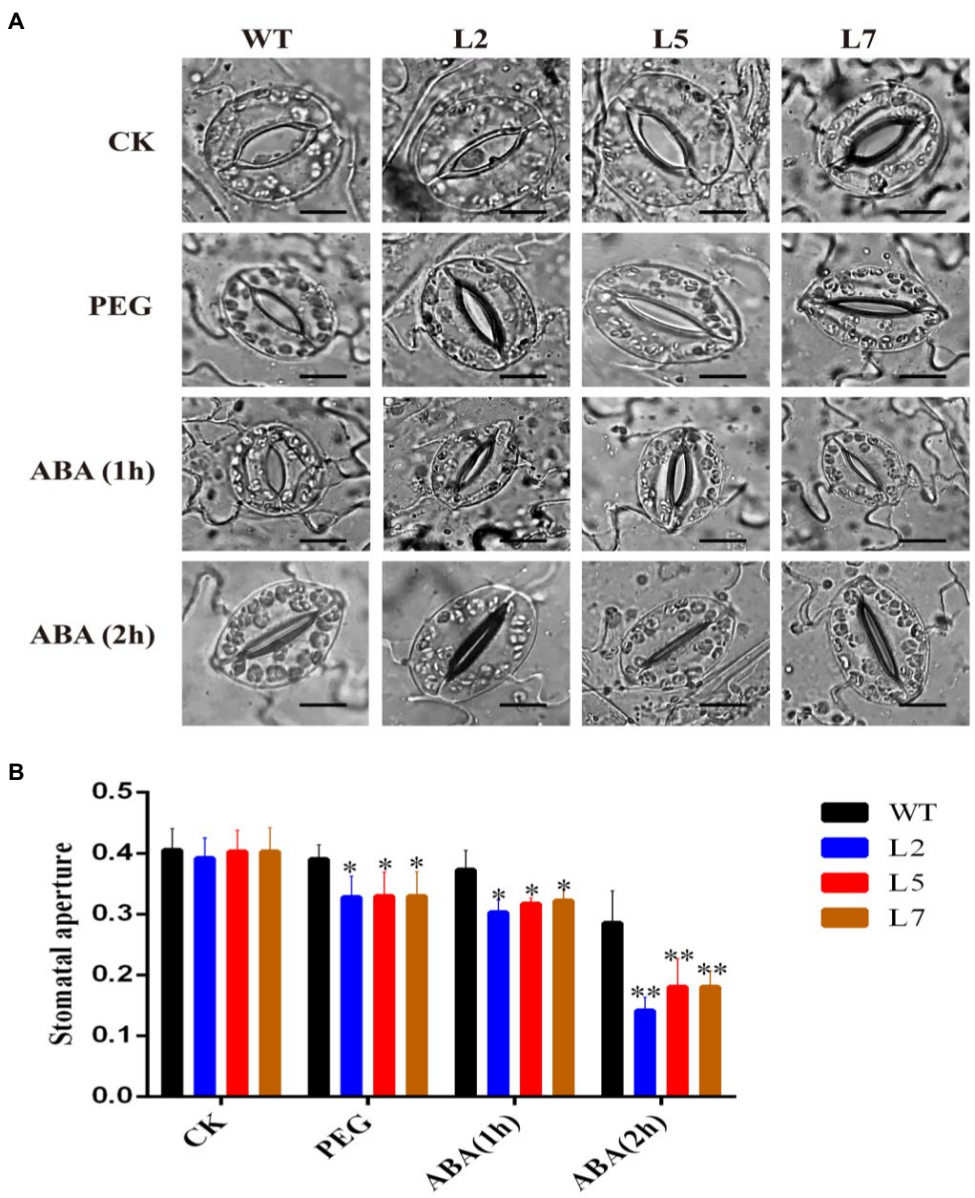
Effects of StNF-YC9 on stomatal aperture detected after PEG and ABA treatment. **(A)** Phenotype of stomatal opening phase in OxStNF-YC9 lines (L2, L5, and L7) and WT lines with PEG and ABA treatment. The scale bar represents 50μm. **(B)** Stomal aperture is represented by the ratio of width to length of stomata. Data shown are means±SE (*n*=80). The asterisks represent significant differences compared to the control (^*^p<0.05 and ^**^p<0.01).

To investigate whether StNF-YC9 participates in PEG and ABA-induced stomatal closure, the leaf stomata size of 28-day-old OxStNF-YC9 lines was compared to WT. After treatment with PEG and exogenous ABA, the stomatal aperture size in OxStNF-YC9 lines was less than that in WT, while no significant difference was observed without treatment (Figures 6A,B). These results suggested that StNF-YC9 participates in the regulation of stomatal aperture under PEG and ABA treatments.

### Overexpression of *StNF-YC9* Improves Drought Stress Tolerance

**Figure 7:**
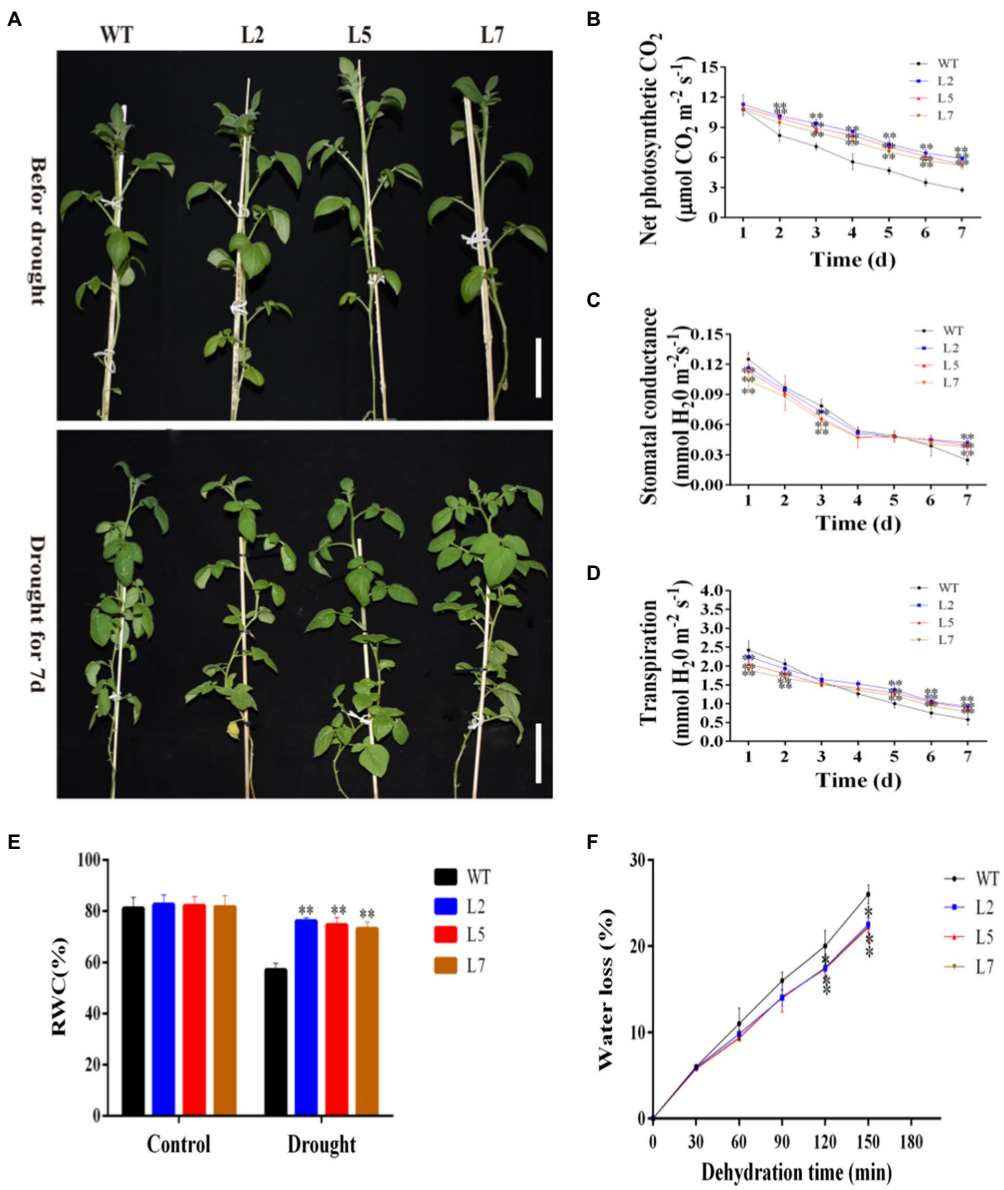
Overexpression of *StNF-YC9* improved drought tolerance after 7days of mild drought. **(A)** Phenotypic differences between WT and OxStNF-YA9 lines (L2, L5, and L7) after 7days of mild drought. The scale bale represents 10cm. **(B)** Net photosynthesis rate relative to drought duration. **(C)** Stomatal conductance relative to drought duration. **(D)** Stomatal conductance relative to drought condition. **(E)** Leaf relative water content under non-stress and drought conditions. **(F)** Water loss from detached leaves. Data are means±SE (*n*=4). Data were analyzed by Student’s *t* tests. The asterisks represent significant differences compared to the control (^*^p<0.05 and ^**^p<0.01).

**Figure 8:**
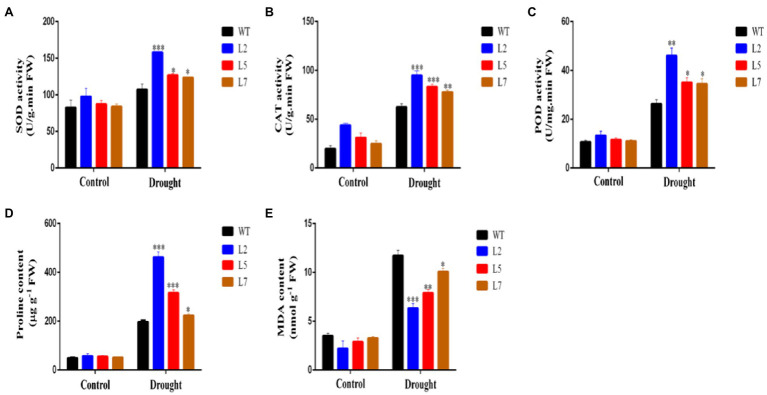
Activity of antioxidant-related enzymes and osmolyte quantification in leaves sampled from plants under well-watered conditions and after 14days of drought. **(A)** SOD activity. **(B)** CAT activity. **(C)** POD activity. **(D)** Proline content. **(E)** MDA content. Data were analyzed by Student’s *t* tests. The asterisks represent significant differences compared to the control (^*^p<0.05, ^**^p<0.01, and ^***^p<0.001).

To elucidate the function of *StNF-YC9* in drought tolerance, water deficit stress was imposed on WT and OxStNF-YC9 lines grown in soil for 28 days old by keeping the soil RWC at 45% (mild drought stress) for 7days. WT plants withered after 7days, while the OxStNF-YC9 did not (Figure 7A). Photosynthesis system-related parameters, including net photosynthesis rate, stomatal conductance, and transpiration rate, were also found to be higher in OxStNF-YC9 lines compared to WT during drought treatment. The net photosynthesis rate in OxStNF-YC9 lines also decreased much more slowly than that in WT during drought treatment (Figure 7B). The stomatal conductance and transpiration rate also decreased, but the reduction in OxStNF-YC9 lines was slower than that in WT (Figures 7C,D). For RWC, there was no significant difference between the WT and OxStNF-YC9 lines in well-watered conditions, but after 7days of drought treatment, the RWC of the WT lines was much lower (Figure 7E). In addition, the water loss rate increased much more slowly in OxStNF-YC9 lines compared to WT plants (Figure 7F). To further investigate the effects of OxStNF-YC9 plants in long drought stress, the OE lines and WT were subjected to 14days of soil RWC at 70% (control) or 45% (mild drought stress). The activity levels of three key antioxidant enzymes, superoxide SOD, CAT, and POD, were measured. As shown in Figures 8A–C, the enzyme activities of SOD, CAT, and POD in OxStNF-YC9 plants were not substantially higher than those in WT under normal conditions, whereas the activities of the three enzymes were significantly higher in the OxStNF-YC9 lines compared to WT during drought treatment. Under normal conditions, there were no significant differences between the WT and OxStNF-YC9 lines in MDA or proline content, whereas the two physiological indicators were significantly higher in the OxStNF-YC9 plants compared to WT plants during drought treatment (Figures 8D,E).

## Discussion

### Evolutionary Analysis and Classification of *StNF-Y* Genes

In the present study, a total of 41 NF-Y members were identified in potato, compared to the amount found in Arabidopsis (36 NF-Y members; Siefers et al., 2009), rice (34 NF-Y members; Yang et al., 2017), and poplar (52 NF-Y members; Liu et al., 2020). In general, the number of *NF-Y* genes identified in these plants is positively correlated with genome size. Protein multiple sequence alignments revealed a conserved region with a S-R-H-…G-G-R-F motif in the C-terminal region of NF-YA subunits (Supplementary Figure S1A), which likely binds the CCAAT box sequence (Laloum et al., 2013). This same conserved amino acid motif was found in both NF-YB and NF-YC, indicating that it is crucial to NF-Y DNA binding activity (Myers and Holt, 2018). It is well-established that high sequence identity between species can be used to infer similar functions (Petroni et al., 2012). To predict potential functions of the *StNF-Y* genes in potato, an unrooted phylogenetic tree was constructed from the protein sequences of all 41 StNF-Ys and 36 AtNF-Ys (Figure 1). As shown in Figure 1, the NF-Ys formed mixed clusters, which contained StNF-Ys and AtNF-Ys. *AtNF-YA7* has been shown to be induced by the drought stress (Leyva-González et al., 2012), and its ortholog, *StNF-YA8*, was also found to be induced by the drought in a previous study (Yang et al., 2016). *AtNF-YB2/3* has been reported to be involved in photoperiodic flowering (Hwang et al., 2016), and its closest neighbor, *StNF-YB20*, plays a role in accelerating the onset of flowering (Xuanyuan et al., 2017). The motif compositions of StNF-Y genes (Figure 2) revealed conserved motifs that corresponded to StNF-YA, StNF-YB, and StNF-YC subgroups. Motifs 2, 5, and 6 were present in the StNF-YA subgroup. Motifs 1, 3, and 5 were found in the StNF-YB subgroup, which is proposed to act as a histone-like and archaeal histone domains (Liu et al., 2020). However, motifs 4 and 9 were unique to the StNF-YC subgroup. Gene structure analysis suggested that *StNF-YA* members shared a similar exon-intron structure (Figure 2), while some *StNF-YB* members had only one exon, which is in agreement with findings in Arabidopsis, *Brassica napus* L. (Liang et al., 2014) and *Gossypium hirsutum* L. (Chen et al., 2018). These results indicate that some *StNF-YB* genes have undergone intron loss during evolution.

### *StNF-Y* Genes Play Vital Roles in Potato Growth, Development and Response to Abiotic Stress

NF-Y transcription factors play crucial roles in plant development and a variety of abiotic stress responses in plants, including salt, drought, heat, and freezing (Petroni et al., 2012; Zhao et al., 2016). As shown in Figure 3A, expression analysis of all *StNF-Y* members in 11 different potato organs and tissues revealed that *StNF-Y* genes in the same evolutionary branch have a similar expression profile, indicating that they may participate in a similar developmental processes. *StNF-YC1/2/5* and *StNF-YB16/20* were highly expressed in all tissues examined, while *StNF-YA8* was mainly expressed in mature tubers and tuber sprouts (Figure 3A). The same gene, *StNF-YA8,* studied previously was reported to be induced by drought stress (Yang et al., 2016). Similarly, *PtNF-YA9* has been demonstrated to regulate seed germination, abiotic stress responses, and plant growth and development (Lian et al., 2018). Based on the available transcriptome data, some *StNF-Y* genes are associated with responses to heat, drought, ABA, and salt stress. For example, *StNF-YC1/2/9* and *StNF-YB16/20* were found to be induced by multiple abiotic stresses (Figure 3B). In addition, qRT-PCR was used to validate the salt stress inducibility of *StNF-YA9/B22/C8* (Figure 4A). *AtNF-YA1*, a paralogous gene of *StNF-YA9*, was also shown to be induced by salt stress and is known to regulate post-germination growth retardation during salt stress (Li et al., 2013). Several *StNF-Y* genes were downregulated by treatment with 20% PEG-6000, except for *StNF-YC8/C9* (Figure 4B). Three *StNF-YB* genes (*StNF-YB19/20/22*) were also expressed at higher levels during dehydration stress (Figure 4C). In maize, overexpression of *ZmNF-YB16* increased tolerance to drought stress and dehydration (Wang et al., 2018).

### Overexpression of StNF-YC9 Enhances Root Length and Drought Tolerance

A number of NF-Y transcription factors have been demonstrated to act as regulators of root development in different plants, and overexpression of PdNF-YB7 in Arabidopsis was shown to increase primary root length (Han et al., 2013). Furthermore, Ballif et al. (2011) reported that overexpression of AtNF-YB2 enhanced primary root elongation. Our results showed that overexpression of StNF-YC9 significantly increased root length compared to WT (Supplementary Figure S4; Supplementary Table S4), which is in agreement with results in Arabidopsis (Palmeros-Suárez et al., 2015). Stomata are formed by two guard cells that regulate CO_2_ input to leaves for photosynthesis and water evaporation (Kim et al., 2010). Stomatal aperture is affected by numerous factors, such as light, CO_2_ concentration, abiotic stress, and phytohormones (Saradadevi et al., 2017). Stomatal aperture changes typically affect water use efficiency and drought tolerance (Lawson and Blatt, 2014; McAusland et al., 2016). We therefore investigated whether StNF-YC9 participated in the regulation of stomatal aperture and found that stomatal aperture was significantly smaller in the OxStNF-YC9 lines compared to WT after PEG and ABA treatment (Figure 6). This is in keeping with previous studies that demonstrated that NF-Y transcription factors were involved in ABA-mediated stomatal closure (Xuanyuan et al., 2017; Lian et al., 2018). In general, photosynthetic rate is decreased by drought stress (Ashraf and Harris, 2013) and our results revealed that the net photosynthesis rate of OxStNF-YC9 reduced less than the rate in WT during drought (Figure 7B). Similarly, Wang et al. (2018) overexpressed ZmNF-YB16 and found that it also enhanced photosynthesis and drought tolerance. It has also been reported that stomatal conductance is positively correlated with transpiration and negatively correlated with drought tolerance (Baloch et al., 2011). In the present study, the stomatal conductance and transpiration rate also decreased during drought, but the reduction in OxStNF-YC9 lines was slower than that in WT (Figures 7C,D). RWC is a critical index that is used to indicate plant tolerance to drought stress (Griffiths et al., 2014). The RWC in OxStNF-YC9 lines was significantly higher than that in WT upon drought stress (Figure 7E). To cope with drought stress, plants also employ antioxidant enzymes, including SOD, CAT, and POD, along with MDA and proline (Madhulika et al., 2015). We found that the activity levels of SOD, CAT, and POD were markedly increased in OxStNF-YC9 lines compared with WT plants under drought stress (Figures 8A–C), which indicated that the antioxidant enzyme system activity is increased in OxStNF-YC9 plants, protecting them from ROS toxicity under drought stress. In addition, OxStNF-YC9 plants exhibited a significant increase in proline, which adjusts osmotic pressure and a decrease in MDA, which is a lipid peroxidation indicator (Figures 8D,E). This further confirmed that overexpression of StNF-YC9 in potato enhanced potato drought tolerance. These findings are in keeping with results in transgenic tobacco, which showed that overexpression of AsNF-YC8 caused enhanced drought and salt stress tolerance by regulating the activities of antioxidant enzymes (Sun et al., 2017). On the contrary, it was recently reported by Chen et al. (2020) that overexpression of SlNF-YA10 in tomato caused a significant increase in MDA content and a significant decrease in POD activity. Our results represent an important first step in understanding the role of StNF-YC9 in potato growth, development, and abiotic stress responses, although the underlying mechanisms of this gene require further study.

## Conclusion

In the present study, a total of 41 *StNF-Y* genes were identified in potato genome. A comprehensive of structural features, phylogenetic analyses, and expression profiles was performed. Overexpression of StNF-YC9 transgenic plants has increased root length and reduced stomatal aperture in potato treated by polyethylene-glycol and abscisic acid. Furthermore, the overexpression of StNF-YC9 increased net photosynthesis, antioxidant (superoxide dismutase, catalase, and peroxidase) system activation, proline contents, and decreased malondialdehyde that enhanced drought tolerance in potato. These results provide insight into understanding evolution of the *StNF-Y* family genes in potato and the potential role in genetic improvement.

## Data Availability Statement

The original contributions presented in the study are included in the article/Supplementary Material, further inquiries can be directed to the corresponding author.

## Author Contributions

HJS and SGL conceived and designed the experiments. SGL, XZ, RM, SYL, XW, and JWY performed the laboratory experiments. SGL, NZ, and HJS performed the data analysis and interpretation. SGL and HJS wrote the paper. All authors contributed to the article and approved the submitted version.

## Funding

This research was sponsored by State Key Laboratory of Aridland Crop Science, Gansu Agricultural University (GSCS-2019-Z03) and the National Natural Science Foundation of China (31860399 and 31960444).

## Conflict of Interest

The authors declare that the research was conducted in the absence of any commercial or financial relationships that could be construed as a potential conflict of interest.

## Publisher’s Note

All claims expressed in this article are solely those of the authors and do not necessarily represent those of their affiliated organizations, or those of the publisher, the editors and the reviewers. Any product that may be evaluated in this article, or claim that may be made by its manufacturer, is not guaranteed or endorsed by the publisher.

## Footnotes

^1^ https://phytozome-next.jgi.doe.gov/

^2^ http://www.ebi.ac.uk/interpro/

^3^ http://pfam.xfam.org/

^4^ https://www.expasy.org/

^5^ http://gsds.cbi.pku.edu.cn

^6^ https://meme-suite.org/meme/

## Supplementary Material

Supplementary Figure S1 Alignment of tomato and *A. thaliana* NF-Y domains of NF-YA **(A)**, NF-YB **(B)**, and NF-YC **(C)**.

Supplementary Figure S2 Chromosomal locations of *StNF-Y* gene family.

Supplementary Figure S3 Analysis of StNF-YC9 overexpression transgenic plant lines.

Supplementary Figure S4 Phenotypes of 14-day-old OxStNF-YC9 lines (L2, L5, and L7) and WT plants.
