# Supplementary material for: Genome-Wide Analysis of NF-Y Genes in Potato and Functional Identification of StNF-YC9 in Drought Tolerance
Source: Front Plant Sci. 2021 Oct 12;12:749688. doi: 10.3389/fpls.2021.749688 (PMC8631771; doi:10.3389/fpls.2021.749688)
Supplement: Supplementary file 1 [file Table_1.docx]

Table S1 The primers used in the present study.

| Primer name | Primer sequence | Purpose |
| --- | --- | --- |
| StNF-YA1-F | TCCTCAACTTTCGGGCCGCATT |  |
| StNF-YA1-R | AGGTCGGCGCAAAGCATGGA |  |
| StNF-YA2-F | TTCTGCGGCGAAGAGAGTCACG |  |
| StNF-YA2-R | TCGCAAAACGCCCTCCAGAAGC |  |
| StNF-YA3-F | CATGATGGTCGGCAGCTGGA |  |
| StNF-YA3-R | TTCCCCTGGGCAACGGCATT |  |
| StNF-YA5-F | GCGGGTGTTCCTTTGCCATCA |  |
| StNF-YA5-R | TCAATGCATGCAAATGGCGTGA |  |
| StNF-YA6-F | TGGTTTCTCCTCGCGTGCCACT |  |
| StNF-YA6-R | TGACGATGCCGGGACTCATGGA |  |
| StNF-YA7-F | GGGGTTCTGGAGGGCGCTTTTT |  |
| StNF-YA7-R | TGCCACCTGCCTGTCGCTTA |  |
| StNF-YA8-F | TGGTCAATCCCTGGGCCGGATTA |  |
| StNF-YA8-R | TGGCGCGAGAGGTGCAAGAATG |  |
| StNF-YA9-F | GCCAGTTGAAGTGAAAGAGGAGCCAAT |  |
| StNF-YA9-R | TGTCGGTGACGCGATTCGTGA |  |
| StNF-YA10-F | CCCTGGGTTGATGCGGGAAA |  |
| StNF-YA10-R | TGGCTGGAGCAGCTGGCTGAAA |  |
| StNF-YB6-F | TGCTTTGCGCCATGGACAGG |  |
| StNF-YB6-R | ATCCCCCTGATAGGGCGTGATGCT |  |
| StNF-YB14-F | TGGCATGATGATGATGGGGAATCA |  |
| StNF-YB14-R | CCCAACATCAGGAAATCGCGAACC |  |
| StNF-YB19-F | CGCAGGGTTGTGAAGGAAAAGC |  |
| StNF-YB19-R | GCAGTTGCGGAGAGGTAATGGATGA |  |
| StNF-YB20-F | TCAGGAGGTCACAACAACGCGAACA |  |
| StNF-YB20-R | TCAGGAGGTCACAACAACGCGAACA |  |
| StNF-YB22-F | TTGCCCCCAGATGTCCGTGTTG |  |
| StNF-YB22-R | TCCCCAAAGCCAAGAACCTCCA |  |
| StNF-YC1-F | GGCATGCCACCACAATCTGCTCA |  |
| StNF-YC1-R | GGTTGTTGCTGCTGGGGCCAAA | qRT-PCR |
| StNF-YC2-F | TGGCCATTCTGATGCTGCTGGTG |  |
| StNF-YC2-R | ATCAGCACGGCTTACCCGACCA |  |
| StNF-YC5-F | GGCATGGTGGGTTCCACAGCAA |  |
| StNF-YC5-R | TCAGCAGCCTGCCAAACGGACT |  |
| StNF-YC8-F | TCCCCAGAAGTCGTCGCTCCTGCTA |  |
| StNF-YC8-R | TGGTGCTGGTGCTGGTGTTGCT |  |
| StNF-YC9-F3 | TGCCGAGGGCCTCCCATACAAT |  |
| StNF-YC9-R3 | GGACGGGGATGCTGGCCATAAA |  |
| StNF-YC9-F1 | ATGGATCAGCAAGGAAATGGAC | Clone of StNF-YC9 gene |
| StNF-YC9-R1 | TTAAGAATCTGATGGAGGTTGCTGTTG |  |
| EF1ɑ-F  EF1ɑ-R | ATTGATGCCCCTGGTCACAG  CATGTTCACGGGTCTGACCA | qRT-PCR potato reference gene |
| StNF-YC9-F1 | ATGGATCAGCAAGGAAATGGAC | Clone of StNF-YC9 gene |
| StNF-YC9-R1 | TTAAGAATCTGATGGAGGTTGCTGTTG |  |
| StNF-YC9-F2 | CGCGGATCCATGGATCAGCAAGGAA | Vector construction of pCAMBIA1300-35S-EGFP- StNF-YC9 |
| StNF-YC9-R2 | GACGTCGACAGAATCTGATGGAGGT |  |
| StNF-YC9-F4 | CGCGGATCCATGGATCAGCAAGGAA | Vector construction of pCAMBIA1300-35S- StNF-YC9 |
| StNF-YC9-R4 | TGGCTGCAGTTAAGAATCTGATGGA |  |
| HPT-F | ATTTGTGTACGCCCGACAGT | Identification of putative transgenic potato |
| HPT-R | GATGTAGGAGGGCGTGGATA |  |
