# Supplementary material for: Genome-Wide Analysis of NF-Y Genes in Potato and Functional Identification of StNF-YC9 in Drought Tolerance
Source: Front Plant Sci. 2021 Oct 12;12:749688. doi: 10.3389/fpls.2021.749688 (PMC8631771; doi:10.3389/fpls.2021.749688)
Supplement: Supplementary file 2 [file Table_2.docx]

**Table S2** Sequence characteristics of potato *StNF-Y* genes

| Gene ID | Gene name | Chromosome location | Number of Intron | Length (AA) | pI | Molecular Weight(kDa) | Instability Index | Aliphatic Index | GRAVY |
| --- | --- | --- | --- | --- | --- | --- | --- | --- | --- |
| PGSC0003DMG400021365 | StNF-YA1 | ch01:2798901 | 4 | 311 | 9.63 | 33.97 | 43.64 | 60.87 | -0.490 |
| PGSC0003DMG400010697 | StNF-YA2 | ch01:4309170 | 4 | 310 | 6.5 | 33.79 | 63.00 | 54.19 | -0.948 |
| PGSC0003DMG400006863 | StNF-YA3 | ch01:65741226 | 4 | 303 | 8.77 | 33.18 | 44.42 | 47.79 | -0.954 |
| PGSC0003DMG400011806 | StNF-YA4 | ch02:13842003 | 4 | 202 | 7.78 | 22.58 | 85.80 | 59.06 | -0.890 |
| PGSC0003DMG401021124 | StNF-YA5 | ch02:28993071 | 4 | 210 | 7.17 | 23.22 | 73.69 | 58.14 | -0.885 |
| PGSC0003DMG400002484 | StNF-YA6 | ch03:61246327 | 4 | 283 | 9.65 | 31.87 | 60.55 | 51.02 | -1.028 |
| PGSC0003DMG400021747 | StNF-YA7 | ch08:34346250 | 2 | 216 | 9.35 | 24.32 | 55.69 | 72.59 | -0.414 |
| PGSC0003DMG400028144 | StNF-YA8 | ch10:56173953 | 3 | 196 | 9.54 | 22.06 | 45.61 | 61.73 | -0.747 |
| PGSC0003DMG400008148 | StNF-YA9 | ch10:58591969 | 4 | 240 | 9.23 | 27.30 | 64.14 | 49.96 | -0.981 |
| PGSC0003DMG400007350 | StNF-YA10 | ch11:37859363 | 4 | 300 | 6.72 | 33.16 | 70.93 | 54.37 | -0.970 |
| PGSC0003DMG400022680 | StNF-YB1 | ch01:58347148 | 0 | 136 | 6.14 | 14.93 | 50.21 | 59.56 | -0.887 |
| PGSC0003DMG400024745 | StNF-YB2 | ch01:77136896 | 0 | 138 | 4.93 | 15.99 | 47.58 | 74.20 | -0.726 |
| PGSC0003DMG401024603 | StNF-YB3 | ch03:54482850 | 4 | 159 | 4.67 | 17.96 | 41.41 | 79.12 | -0.502 |
| PGSC0003DMG400032552 | StNF-YB4 | ch04:5168953 | 1 | 132 | 6.59 | 14.57 | 54.04 | 85.00 | -0.385 |
| PGSC0003DMG400044580 | StNF-YB5 | ch04:53783413 | 0 | 191 | 6.18 | 21.36 | 26.92 | 66.91 | -0.730 |
| PGSC0003DMG400030986 | StNF-YB6 | ch05:8408839 | 0 | 135 | 5.34 | 15.02 | 40.43 | 72.96 | -0.530 |
| PGSC0003DMG400013502 | StNF-YB7 | ch05:8570774 | 0 | 179 | 4.85 | 20.20 | 60.02 | 70.28 | -0.548 |
| PGSC0003DMG400013503 | StNF-YB8 | ch05:8582170 | 0 | 161 | 4.72 | 18.18 | 52.52 | 61.74 | -0.622 |
| PGSC0003DMG402013505 | StNF-YB9 | ch05:8599340 | 1 | 224 | 4.91 | 25.03 | 50.54 | 54.42 | -0.710 |
| PGSC0003DMG401013505 | StNF-YB10 | ch05:8599340 | 0 | 189 | 4.73 | 21.37 | 46.08 | 62.43 | -0.583 |
| PGSC0003DMG400034306 | StNF-YB11 | ch05:14237899 | 0 | 198 | 4.73 | 21.81 | 38.78 | 85.20 | -0.243 |
| PGSC0003DMG400014643 | StNF-YB12 | ch06:566943 | 0 | 178 | 5.51 | 20.47 | 41.83 | 73.93 | -0.858 |
| PGSC0003DMG400004076 | StNF-YB13 | ch06:51052551 | 4 | 158 | 4.67 | 17.67 | 47.04 | 74.75 | -0.544 |
| PGSC0003DMG400022172 | StNF-YB14 | ch07:55488546 | 0 | 185 | 5.7 | 20.43 | 35.02 | 61.68 | -0.769 |
| PGSC0003DMG400022277 | StNF-YB15 | ch07:55543376 | 1 | 173 | 9.32 | 19.50 | 43.45 | 72.77 | -0.461 |
| PGSC0003DMG400001733 | StNF-YB16 | ch09:6568240 | 1 | 140 | 7.74 | 15.51 | 56.59 | 81.57 | -0.424 |
| PGSC0003DMG400011385 | StNF-YB17 | ch09:52280038 | 0 | 143 | 6.65 | 16.36 | 36.63 | 72.24 | -0.836 |
| PGSC0003DMG400013302 | StNF-YB18 | ch11:203438 | 0 | 120 | 7.02 | 13.82 | 54.04 | 79.67 | -0.455 |
| PGSC0003DMG400026357 | StNF-YB19 | ch11:4041617 | 2 | 172 | 4.91 | 19.31 | 60.06 | 79.94 | -0.823 |
| PGSC0003DMG400004299 | StNF-YB20 | ch12:6260341 | 0 | 158 | 5.33 | 16.68 | 41.32 | 50.70 | -0.813 |
| PGSC0003DMG400016753 | StNF-YB21 | ch12:23597738 | 0 | 198 | 5.96 | 22.45 | 47.47 | 65.91 | -0.785 |
| PGSC0003DMG400023065 | StNF-YB22 | ch12:43500954 | 4 | 155 | 4.64 | 17.44 | 39.27 | 83.03 | -0.474 |
| PGSC0003DMG400008989 | StNF-YC1 | ch01:62006821 | 0 | 256 | 5.87 | 28.35 | 66.60 | 74.06 | -0.471 |
| PGSC0003DMG400003296 | StNF-YC2 | ch01:74443838 | 3 | 276 | 5.35 | 30.81 | 58.77 | 60.04 | -0.957 |
| PGSC0003DMG400039636 | StNF-YC3 | ch03:50526085 | 0 | 148 | 4.89 | 16.68 | 57.35 | 74.46 | -0.557 |
| PGSC0003DMG400015260 | StNF-YC4 | ch03:50535042 | 0 | 154 | 4.94 | 17.60 | 45.57 | 72.86 | -0.652 |
| PGSC0003DMG402015259 | StNF-YC5 | ch03:50559361 | 1 | 230 | 4.97 | 25.26 | 69.57 | 69.30 | -0.501 |
| PGSC0003DMG400015177 | StNF-YC6 | ch03:51135062 | 0 | 253 | 5.10 | 27.67 | 64.49 | 71.34 | -0.260 |
| PGSC0003DMG400029106 | StNF-YC7 | ch05:13620719 | 0 | 141 | 9.33 | 15.67 | 61.38 | 88.58 | -0.482 |
| PGSC0003DMG400027390 | StNF-YC8 | ch11:43724874 | 3 | 293 | 4.97 | 32.28 | 51.41 | 67.30 | -0.832 |
| PGSC0003DMG400003357 | StNF-YC9 | ch12:33853117 | 0 | 252 | 5.87 | 28.04 | 63.84 | 69.76 | -0.600 |
