# Supplementary material for: Genome-Wide Analysis of NF-Y Genes in Potato and Functional Identification of StNF-YC9 in Drought Tolerance
Source: Front Plant Sci. 2021 Oct 12;12:749688. doi: 10.3389/fpls.2021.749688 (PMC8631771; doi:10.3389/fpls.2021.749688)
Supplement: Supplementary file 3 [file Table_3.docx]

**Table S3** Analysis and distribution of conserved motifs in potato StNF-Ys.

|  | E-value | width | | Best possible match |
| --- | --- | --- | --- | --- |
| 1 | 1.6e^-347^ | 29 | | KISKDAKETVQECVSEFISFITGEANDKC |
| 2 | 1.4e^-168^ | 15 | | DYIEPLKPYLHRYRE |
| 3 | 1.2e^-216^ | 21 | | REKRKTINGEDVLWALTRLGF |
| 4 | 2.9e^-160^ | 50 | | NHQLPLARIKKIMKADEDVRMIAAEAPVLFAKACELFIQELTLRSWLHAE |
| 5 | 3.5e^-144^ | 15 | | FVPIANYHRIMKRRLPRAK |
| 6 | 2.3e^-069^ | 21 | | LHALRRARGSGGRFLNTKKKB |
| 7 | 2.1e^-043^ | 28 | | HHLGYPPPMGNGDMQGDASNGSTSQCAV |
| 8 | 2.5e-040 | 41 | | DDGGECGSLIGESLLKRPMVDTASNCNITPYHQPPNFPMAH |
| 9 | 5.6e-035 | 29 | QQQQQQLQQQLQMFWTNQRQEIEHINDFK | |
| 10 | 5.3e-025 | 28 | EYIEEVYAAYEQHKLETVDTVRAGKCSN | |
