# Supplementary material for: Genome-Wide Analysis of NF-Y Genes in Potato and Functional Identification of StNF-YC9 in Drought Tolerance
Source: Front Plant Sci. 2021 Oct 12;12:749688. doi: 10.3389/fpls.2021.749688 (PMC8631771; doi:10.3389/fpls.2021.749688)
Supplement: Supplementary file 4 [file Table_4.docx]

**Table S4** The morphological characterization of the overexpression of *StNF-YC9* in potato

| Line | Plant height (cm) | Root length (cm) | Fresh weight (g) | root fresh weight | Root - shoot ratio(%) |
| --- | --- | --- | --- | --- | --- |
| WT | 8.17 ± 0.38bc | 4.77 ± 0.32b | 0.1448 ± 0.02b | 0.0530 ± 0.04b | 51.64 ± 0.05a |
| L2 | 7.67 ± 0.55c | 7.27 ± 0.25a | 0.1673 ± 0.02b | 0.0590 ± 0.05b | 55.19 ± 0.06a |
| L5 | 8.43 ± 0.23b | 6.83 ± 0.45a | 0.3701 ± 0.09a | 0.1283 ±  0.04a | 53.44 ± 0.06a |
| L7 | 9.50 ± 0.20a | 7.16 ± 0.59a | 0.4136 ± 0.06a | 0.1430 ±  0.06a | 53.13 ± 0.03a |

Note: The different letters mean significant difference (*P* < 0.05).
