# Supplementary figures and images for: Genome-Wide Analysis of NF-Y Genes in Potato and Functional Identification of StNF-YC9 in Drought Tolerance
Source: Front Plant Sci. 2021 Oct 12;12:749688. doi: 10.3389/fpls.2021.749688 (PMC8631771; doi:10.3389/fpls.2021.749688)

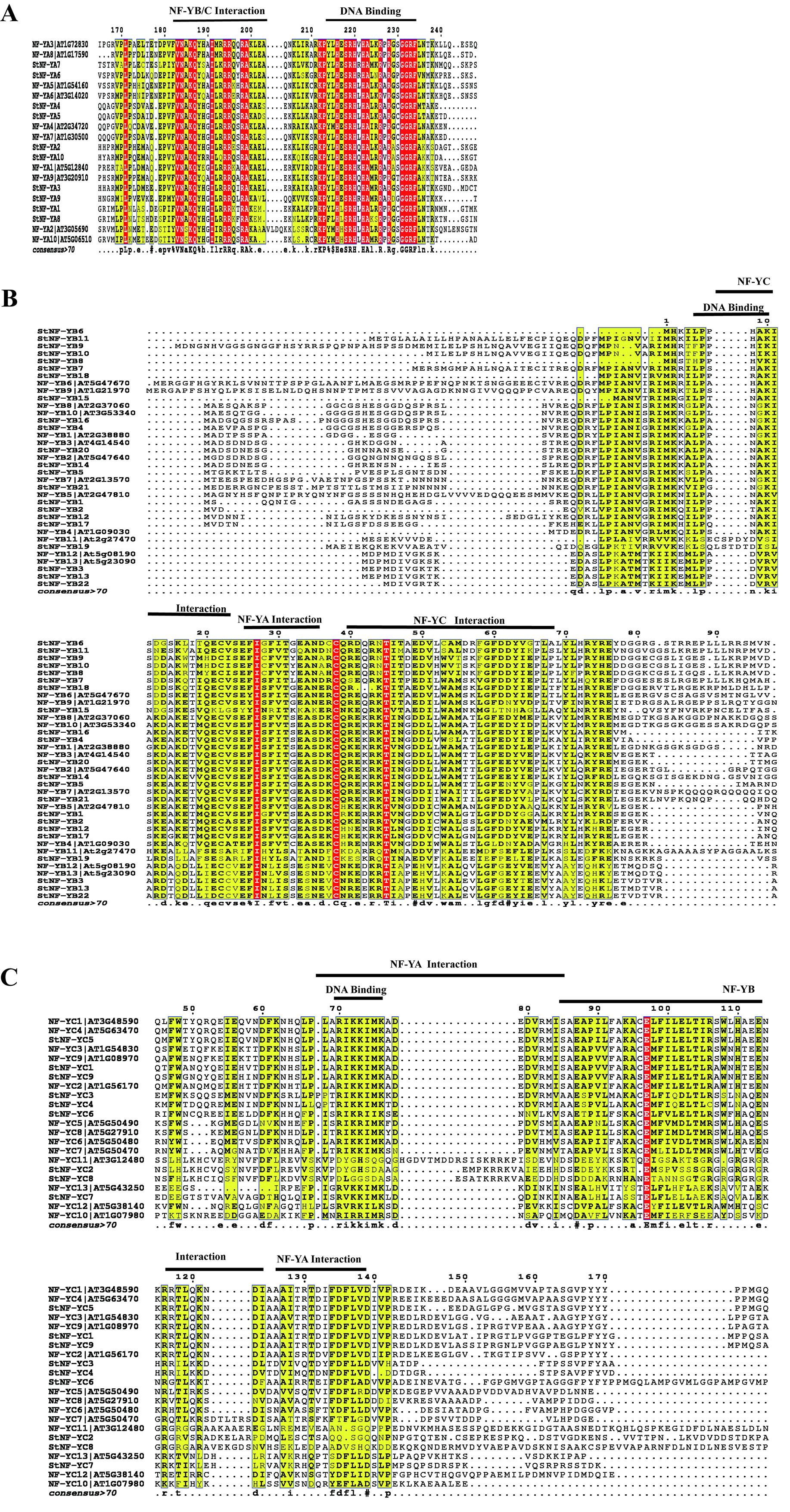

Supplement: Supplementary Figure S1 — Alignment of tomato and A. thaliana NF-Y domains of NF-YA (A), NF-YB (B), and NF-YC (C). [file Image_1.TIF]

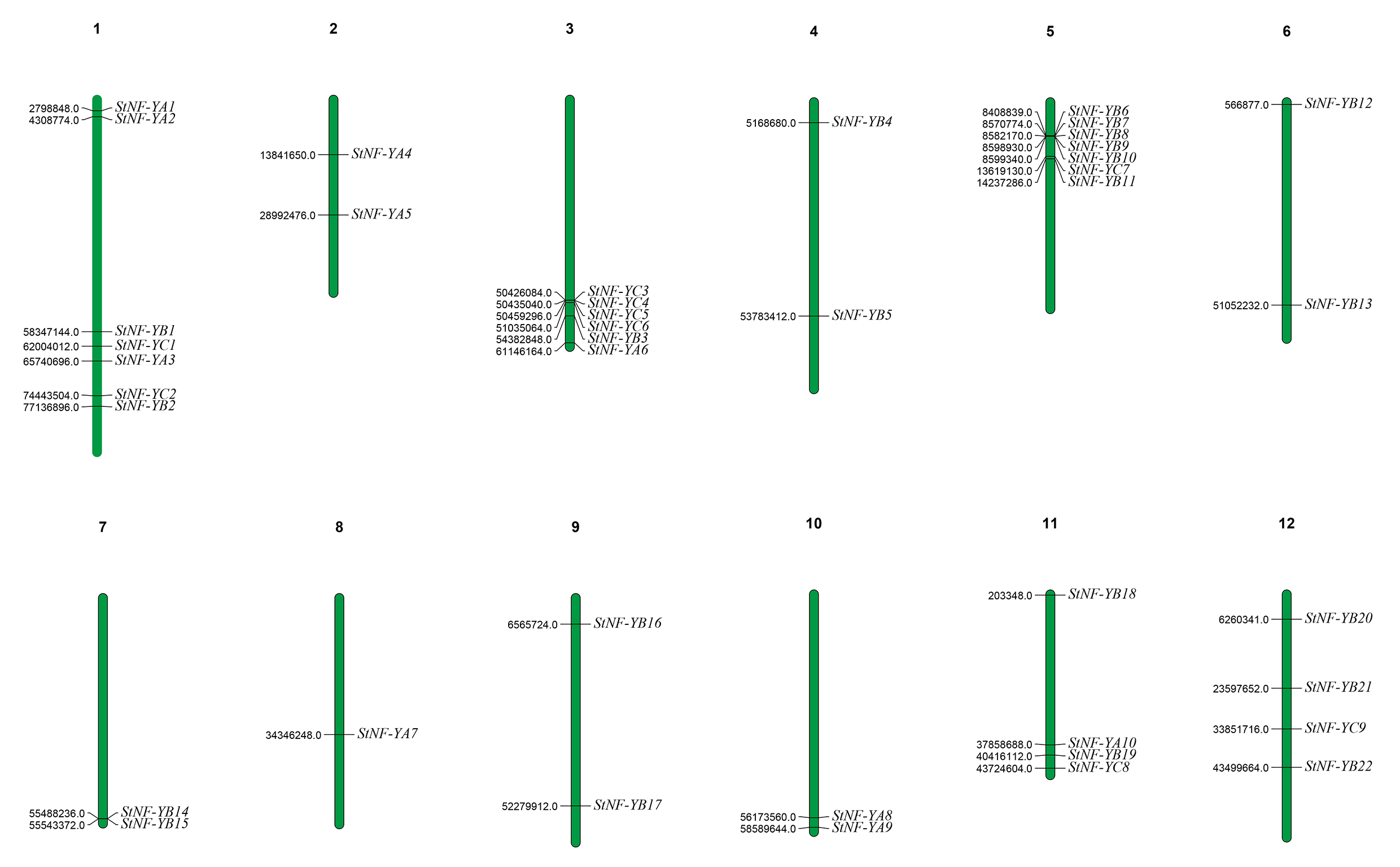

Supplement: Supplementary Figure S2 — Chromosomal locations of StNF-Y gene family. [file Image_2.TIF]

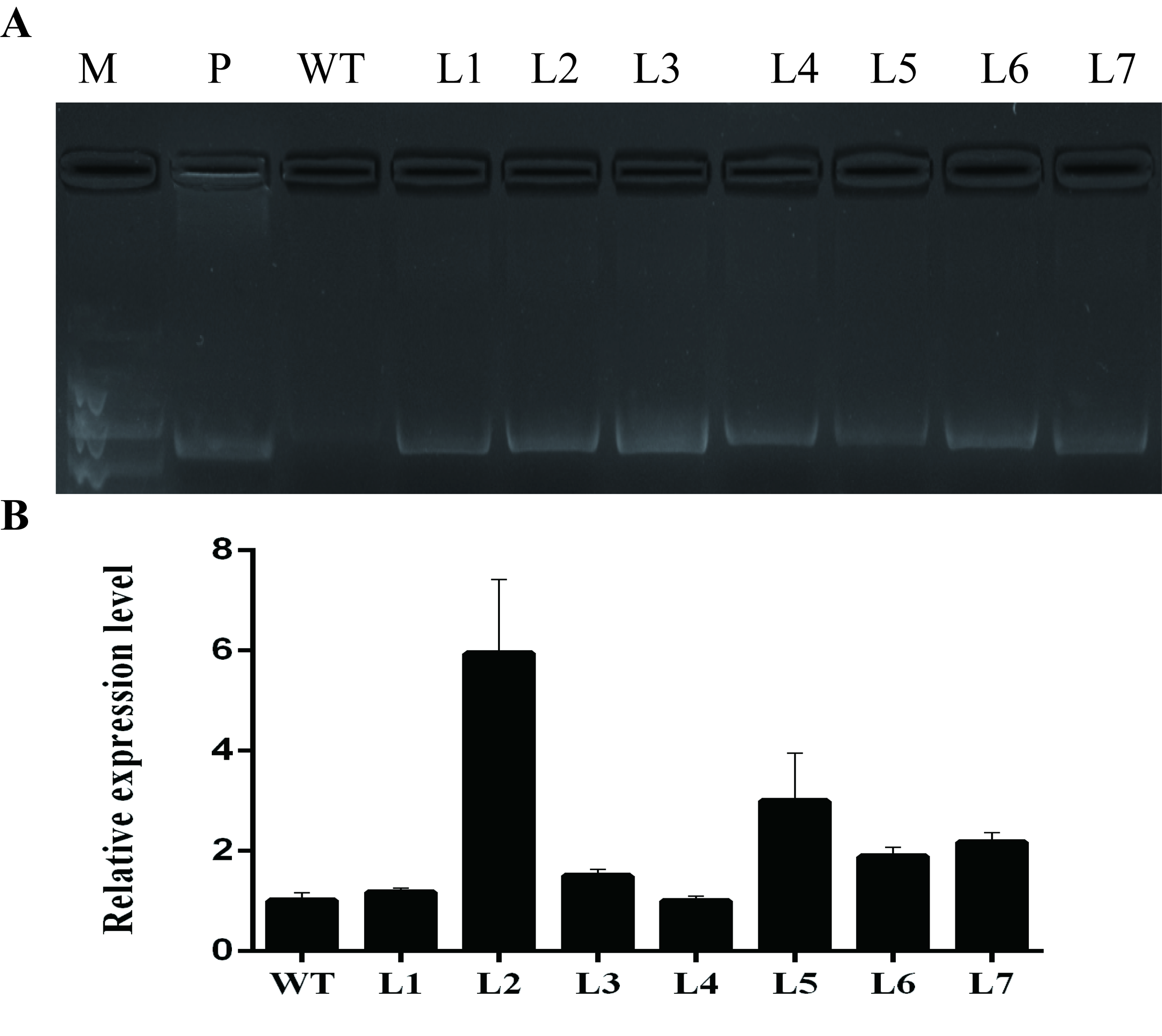

Supplement: Supplementary Figure S3 — Analysis of StNF-YC9 overexpression transgenic plant lines. [file Image_3.TIF]

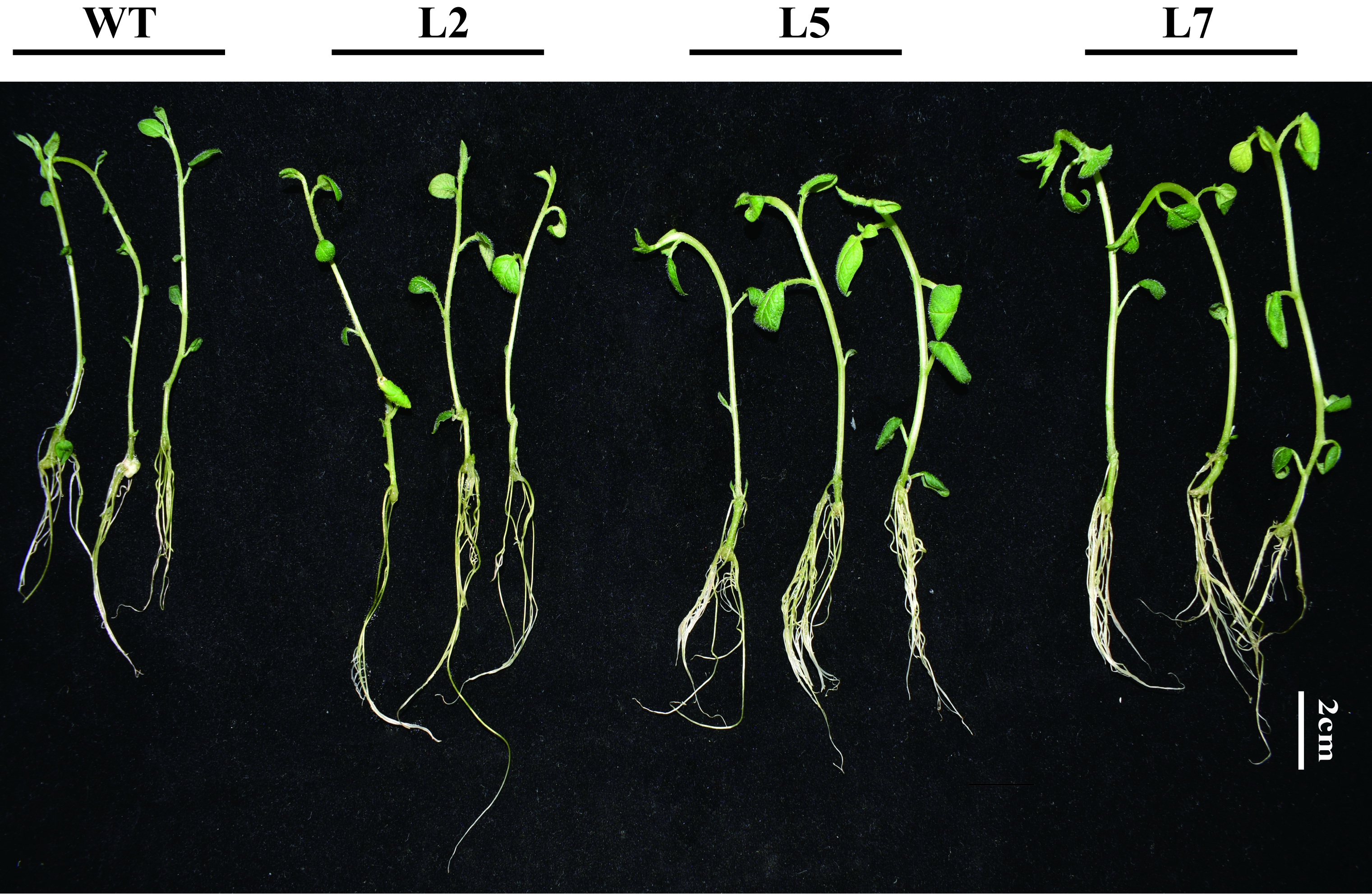

Supplement: Supplementary Figure S4 — Phenotypes of 14-day-old OxStNF-YC9 lines (L2, L5, and L7) and WT plants. [file Image_4.TIF]
